# Extreme Premature Small for Gestational Age Infants Have Appropriate Catch-up Growth at Term Equivalence Compared with Extreme Premature Appropriate for Gestational Age Infants

**DOI:** 10.4274/jcrpe.galenos.2018.2018.0162

**Published:** 2019-02-20

**Authors:** Sze May Ng, Donatella Pintus, Mark A. Turner

**Affiliations:** 1 University of Liverpool, Institute of Translational Medicine, Department of Women’s and Children’s Health, Liverpool, UK; 2 Southport and Ormskirk Hospital NHS Trust, Department of Paediatrics, Southport, UK

**Keywords:** Small for gestational age, prematurity, growth

## Abstract

Recent studies have shown that small for gestational age (SGA) term infants undergo catch-up growth during infancy but there is limited studies on early growth outcomes of extreme premature SGA infants. The aim of this study was to compare factors associated during birth in extremely premature infants less than 28 weeks’ gestation who were born SGA (<10^th^ percentile for gestational age) with those who were born appropriate-for-gestational age (AGA) (10^th^-89^th^ percentile) and to determine whether there was catch-up growth at term equivalence. One hundred fifty-three extreme premature infants (89 males) born below 28 weeks’ gestation were prospectively recruited. All infants had auxological measurements undertaken and prospective data on pregnancy, maternal factors, perinatal and postnatal data obtained. SGA infants at birth had significantly higher Clinical Risk Index for Babies scores and mortality, lower birth weight, smaller head circumference, smaller mid arm circumference and shorter leg length at time of birth compared with AGA infants. However, at term equivalence, weight and leg length of were not significant between AGA and SGA infants born at extreme prematurity. Our study shows that extreme premature SGA infants have appropriate catch-up growth by the time they reach term equivalence suggesting that postnatal nutrition and care are important determinants of catch-up growth in SGA infants.

**What is already known on this topic?**

Small for gestational age (SGA) infants are at risk of impaired postnatal growth. Majority of SGA infants born at term will have catch-up growth by 2 years of age but there are limited studies on extreme premature SGA infants.

**What this study adds?**

Extremely premature small for gestational age infants born less than 28 week’s gestation have appropriate catch-up growth by the time they reach term equivalence suggesting that postnatal nutrition and care are important determinants of catch-up growth in these infants.

## Introduction

Small for gestational age (SGA) is a term most commonly used to indicate infants born with a weight below the 10^th^ percentile for their gestational age. Infants born SGA are at increased risk of perinatal morbidity, persistent short stature and metabolic alterations in later life (1). Infants born SGA with low birth length who do not achieve catch-up growth by the age of two years have a 7-fold higher relative risk of short stature than children born at normal size (2) and this risk is further increased by extreme prematurity (3). While the vast majority of SGA infants do show catch-up growth by 2 years of age, one in 10 does not (4,5). SGA infants who fail to catch-up and do not reach their target height range, remain short throughout childhood (6,7) and approximately 10% of SGA children will continue to further fall below the 3^rd^ percentile of height into adulthood (8,9). Intrauterine and neonatal growth failure of very low birth weight SGA and premature infants may also have long-term implications for adult health with risk of future adverse metabolic outcomes (8,10,11).

Accordingly, the aim of this study was compare factors associated during birth in extremely premature infants who were born SGA (<10^th^ percentile for gestational age) with those who were born appropriate-for-gestational age (AGA) (10^th^-89^th^ percentile) and to determine whether there was catch-up growth at term equivalence, defined in our study at 36 weeks corrected gestational age (CGA).

## Methods

In our study 153 extreme premature infants (89 males) born less than 28 weeks’ gestation were prospectively recruited from the TIPIT study and the infants were randomised according to trial protocol (12,13). The following were excluded: infants born to mothers with known thyroid disease or on anti-thyroid medications or amiodarone, and infants with major congenital or chromosomal abnormalities. This study is part of a post-hoc analysis to look at comparisons between babies born SGA versus AGA carried out at the Neonatal Intensive Care Unit of Liverpool Women’s Hospital, UK. All infants had auxological measurements undertaken and prospective data on pregnancy, maternal factors, perinatal and postnatal data obtained. Ultrasound assessment of the thyroid gland at 36 weeks’ CGA were also undertaken. Auxological measurements and data of SGA infants were compared with AGA infants to compare outcome measures at term equivalence.

### Auxology Measurements

### a) Mini-knemometry

**Figure 1:**
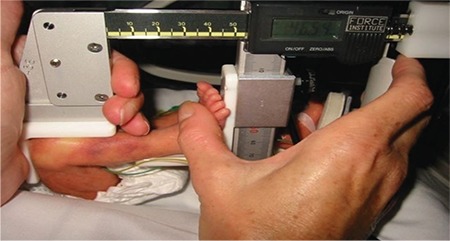
Knemometry measurement

The mini knemometer was used to measure the length of the lower leg of the infants and to determine short term growth in SGA and AGA premature babies. This device has been validated as an accurate device for the detection of small growth spurts that in neonates occur within days (14). The mini knemometer measures the distance from the bottom of the heel to the top of the knee in neonates with the knee flexed at an approx. 90 degrees angle (Figure 1). In order to avoid measurement bias and to develop a standardised method for measuring the infants leg length using this device, a 4-week learning period was required to provide reliable results. Lower leg length measurements of 6 preterm infants were taken using the mini knemometer followed by repeated measurements within a day period for each infant. The acquired mean technical error (mean standard deviation of 5 sequential readings) in all 6 studied infants was 0.15 mm (Table 1).

### b) Mid arm circumference (MAC)

The MAC for each infant was measured using a normal nonstretch measuring tape and measurements were rounded to the nearest 0.1 cm. The measuring point called the mid-point of the upper arm was located in the mid upper arm half way between the tip of the shoulder blade and tip of the elbow. All infants were undressed during these measurements.

### c) Head circumference

The occipitofrontal circumference for each infant was measured using a normal non-stretch measuring tape and measurements were rounded to the nearest 0.1 cm.

### Statistical Analyses

Distributions of continuous outcomes were checked. P values were calculated using a t-test or Mann-Whitney U test as appropriate. Comparisons of SGA and AGA infants were made using univariate analyses. Statistical Package for the Social Sciences 21.0 was used in the data analysis. The study was approved by North West Research Ethics Committee (reference number 07/MRE08/37).

### Consent

The study was approved by North West Research Ethics Committee (reference number: 07/MRE08/37) and by the Medicines for Human Regulatory Agency. The parents of each potentially eligible baby were informed of the study’s objectives and overall requirements after birth when the baby had achieved respiratory and haemodynamic stability. The Investigator explained the study fully to the patient’s parent(s)/guardian(s) using the patient information leaflet. The parent/guardian was then given at least 12 hours to consider the study. If a parent/guardian was willing for the patient to participate in the study written informed consent was obtained.

## Results

One hundred fifty-three infants were recruited to the study. The median gestational age at birth was 26.2±1.0 and mean birth weight was 893.8±178.1 grams. This study found that SGA infants at birth had significantly higher Clinical Risk Index for Babies scores and mortality, lower birth weight, smaller head circumference, smaller MAC and shorter leg length at time of birth compared with AGA infants. However, at term equivalence 36 weeks’ gestation, weight and leg length of SGA and AGA infants were not significant (Table 2).

## Discussion

SGA is a sign of growth failure due to maternal factors such as poor nutrition, chronic disease and infections (5,15), as well as potential environmental toxins (e.g. smoking and alcohol consumption) (16) and paternal factors including diabetes may also contribute to being born SGA (17). The aetiology of most SGA births remains unknown; however, several factors involving the foetus and placenta have been evaluated (15,18). Among these causes, lack of nutritional supply to the foetus is believed to be the primary cause of reduced foetal growth (19). In a Swedish population-based study, highest rates of mortality were observed in extremely premature SGA infants (15). This was in concordance with the results shown in our study.

General postnatal growth pattern can be divided into three phases: infancy, childhood, and puberty and failure of growth in any of these phases can reduce growth potential and eventually cause adult short stature (19). Studies have shown that SGA born neonates may experience a compensatory growth spurt or catch-up growth in infancy and childhood, however the timing of this growth spurt is not well described (20). Catch-up growth of infants born SGA mainly occurs from 6 months to 2 years and approximately 85% of SGA children will have catch-up by two years of age (21), however, babies born prematurely who are SGA may take around four years to achieve catch-up growth (22). The majority of infants born SGA show catch-up growth during the first few months of life followed by a normal pattern of development (19,23). In our study, SGA infants showed appropriate catch-up growth by the time they reached 36 weeks’ CGA suggesting that early postnatal nutrition and care were important determinants of catch-up growth in SGA infants. 

The thyroid hormones are essential for normal growth and development of the foetus and deficiency of thyroid hormones perinatally can impairs growth of the infant and compromises its adaptation to extrauterine life (24). Our results have not found any significant difference in either the maternal or infant thyroid status.

It is generally recognised that children born SGA have a significantly higher risk of short stature in adulthood than children born AGA at normal size, with a possible explanation that the programming of the endocrine axes occurs during critical phases of foetal development affected by intrauterine growth retardation (6). Accurate gestational dating and measurements of birth weight and length are important in identifying SGA infants. We recommend that comprehensive pregnancy, maternal, perinatal, and immediate postnatal data be obtained to help confirm diagnosis although often the cause of SGA remains unclear.

### Limitations of the Study

Limitations of this study is the small sample size of this cohort of extremely premature infants born less than 28 weeks’ gestation and the lack of data on maternal size or maternal glycaemic indices which could be potential confounding factors. Due to their extremes of viability, we were unable to obtain blood sampling for growth hormone factors and body size measurement of these infants were not feasible. The authors believe that the mini knemometer is a practical and novel method used to measure the length of the lower leg of the infants and to determine short term growth these extremely premature babies. The authors also acknowledge that many of extremely premature infants is unlikely to have appropriate catch-up growth by the time they reach term equivalence and factors associated with growth will require further research in a larger population group, which would be best performed as a multi-centered study.

## Conclusions

The mechanism of catch-up growth in premature SGA infants remains unclear (11). Our study shows that extremely premature SGA infants have appropriate catch-up growth by the time they reach term equivalence suggesting that postnatal nutrition and care are important determinants of catch-up growth in SGA infants. We recommend that early surveillance in a growth clinic for those without catchup is advisable and neurodevelopment evaluation and interventions are warranted in at-risk children who fail to catch-up.

## Figures and Tables

**Table 1:**
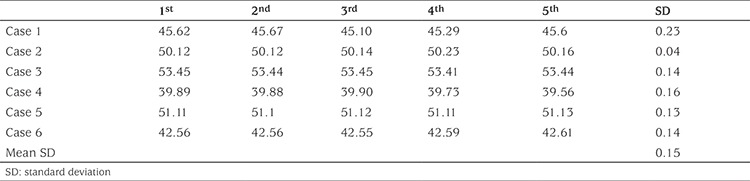
Reliability of mini-knemometry

**Table 2:**
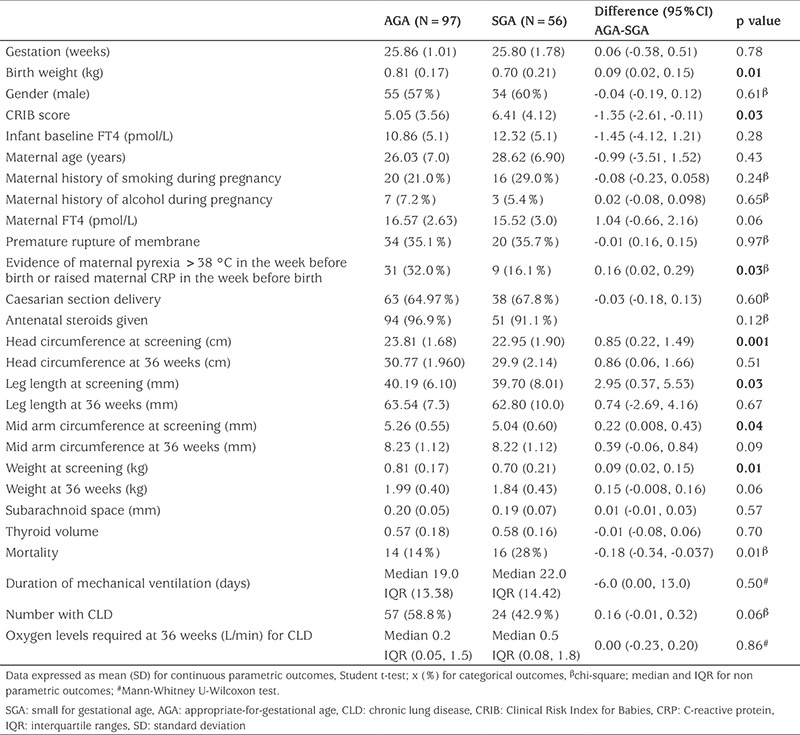
Comparative variables of small for gestational age and appropriate-for-gestational age infants
